# Cancer fertility preservation: a report from a Brazilian social program

**DOI:** 10.5935/1518-0557.20190089

**Published:** 2020

**Authors:** Caroline Z Berton, Caroline Brogliato, Ivan H Yoshida, Laura T Vellez, Claudia H Suganuma, Emerson B Cordts, Gabriel S Conceição, Caio P Barbosa

**Affiliations:** 1 Instituto Idéia Fértil de Saúde Reprodutiva, Santo André, SP, Brasil

**Keywords:** oncological fertility preservation, oocyte preservation, vitrification, social program

## Abstract

**Objective:**

To present clinical and laboratory data of a Brazilian social program for cancer fertility preservation.

**Methods:**

We carried out a descriptive observational study between July 2011 and December 2018. 246 patients were included from a social program in a private assisted reproduction clinic in Santo André/Brazil for oocyte cryopreservation before starting oncological treatment.

**Results:**

246 cancer patients resorted to fertility preservation before initiating cancer treatment. These were diagnosed with 27 different types of cancer, and the breast type is the most prevalent. 2528 MII oocytes (mean of 10.3 oocytes per patient) were vitrified. Four patients thawed their oocytes to submit *in vitro* fertilization, three had embryos transferred and one achieved pregnancy.

**Conclusion:**

Preservation of fertility offers patients, especially at reproductive age, a viable way to perform their cancer treatment without compromising future gestation. It is important that professionals duly counsel oncological patients so, if they wish, they can have the possibility to guarantee her fertility preserved.

## INTRODUCTION

Cancer incidence in children, adolescents and young people has had a considerable increase since 1970 (Siegel *et al*., 2018; INCA, 2018). The therapeutic innovations and advances in the surgical treatment of hematopoietic cells, chemotherapies and radiotherapy have brought about an improvement on the survival of pediatric and young adult patients (Takai, 2018; Salih *et al*., 2015).

Therapeutic strategies such as chemotherapy, radiotherapy or the combination of those two are highly toxic to the gonads (Takai, 2018; Donnez & Dolmans, 2017). The diagnosis of cancer when performed in the early stages of the disease, gives the patient the possibility to undergo fertility preservation techniques before starting any other treatment and, in the future, the intention to have a family with her own gametes is not interrupted (Lunardi *et al*., 2013). The most known fertility preservation techniques are ovarian and testicular tissue cryopreservation, embryo cryopreservation and gametes cryopreservation. That, coupled with *in vitro* fertilization techniques, can result in gestation after the end of the cancer treatment (Hartt, 2014).

In order to overcome the risk of ovarian failure or iatrogenic infertility that treatments used to fight cancer may cause, it is recommended that patients perform cryopreservation of their gametes. The oocytes had larger size, higher water concentration and unique chromosome arrangement, which makes their cryopreservation a challenge (Baka *et al*., 1995). Vitrification is one of the most significant achievements in the field of assisted human reproduction, becoming quite widespread (De Munck & Vajta, 2017). The high initial concentration of cryoprotectants and ultra-fast freezing avoids the undesired formation of intra- and extracellular ice crystals that the slow freezing technique presents (Liang & Motan, 2016; Papadopoulos *et al*., 2002). Rienzi *et al*. (2017) reported that, in terms of clinical outcomes, vitrification was superior to slow freezing. The cryopreservation of oocytes by vitrification is a safe and efficient method, being an alternative to preserve fertility in women submitted to oncological treatments.

The goal of this study was to report on clinical and laboratorial data from a Brazilian social program of fertility preservation in oncological patients.

## MATERIAL AND METHODS

A descriptive observational study was carried out at Instituto Idéia Fertil - Santo André during the period between July 2011 and December 2018. A total of 246 patients sought social services for oocyte cryopreservation before initiating cancer treatment.

We included in this study patients undergoing oncologic treatment, whom had their oocytes frozen, aiming at preserving their fertility.

We excluded oncological patients who chose to cryopreserve their embryos instead of oocytes for future gestation.

Patients who cryopreserved oocytes were of different ages and were affected by several types of cancer.

## RESULTS

There were twenty-seven types of cancer in the patients who cryopreserved their oocytes. The most prevalent were breast cancer (62.6%), ovarian cancer (10.1%), Hodgkin's lymphoma (6.9%), gastrointestinal cancer (3.2%), colon cancer and thyroid cancer, invasive ductal cancer and teratoma (1.6% each). The youngest patient who underwent oncological fertility preservation was 14 years old and the oldest was 47 years old. The mean age of these patients was 31±5.6 years. 2528 oocytes were cryopreserved, with a mean of 10.3 oocytes per patient.

Only four patients returned to the service to continue their *in vitro* fertilization treatment. Their oocytes were thawed and submitted to intracytoplasmic sperm injection technique (ICSI). Of these, three had their embryos transferred on the 3^rd^ day of development and one patient had a positive pregnancy outcome.

## DISCUSSION

Fertility preservation enables women with low ovarian reserve, old age, menopaused or who need to postpone motherhood for personal reasons to achieve the dream of becoming mothers without resorting to donating oocytes. This technique has given hope to a specific group of patients: young women in reproductive age, diagnosed with cancer and with a good chance of surviving, since the treatment of the disease may compromise their chances of future pregnancy. According to Kusnetzoff (1997), experiencing infertility can provoke negative feelings (trauma, jealousy, envy) and intense stress related to the frustration of not being able to conceive. This emotional state can have a counterproductive impact during and after cancer treatment. Infertility can bring irreparable emotional and social repercussions that disrespect the autonomy and desire of the individual to constitute her family. In these cases, the future possibility of having a baby can bring safety and hope to the patient and can become a reality through fertility preservation techniques.

Many women are unaware of the consequence that chemotherapy treatment may have on fertility (Henry *et al*., 2014; Peate *et al*., 2011; Jukkala *et al*., 2010). The onset of treatment to preserve fertility as soon as the cancer is discovered is critical (Baynosa *et al*., 2009; Loren *et al*., 2013). Oocyte retrieval should preferably be performed prior to the beginning of chemotherapy. However, it is necessary for the patient to undergo an ovarian stimulation lasting at least 10 to 14 days, and many patients cannot wait to start their cancer treatment (Takai, 2018).

The extension of damage induced by chemotherapy and radiation varies from case to case, leading to sterility or partial damage. The gonad toxicity of the treatments is dependent on the ovarian reserve, age of the patient at the time of treatment, doses and duration of the therapies administered. The counselling to preserve one’s fertility should be offered to all patients (Donnez & Dolmans, 2017).

To date, there are not many studies describing success rates of pregnancy and live birth following cryopreservation of oocytes and subsequent *in vitro* fertilization treatment, so ovulation induction should be recommended in cases which patient safety is not compromised (Shapira *et al*., 2015).

## CONCLUSION

This study shows that fertility preservation for cancer patients, especially those in reproductive age; provide them a viable method to preserve their fertility. It is important that scientific advances and oocyte cryopreservation be known to professionals who advise oncology patients to perform appropriate treatments and ensure preservation of their fertility.
